# Mapping the Nonreciprocal Micromechanics of Individual Cells and the Surrounding Matrix Within Living Tissues

**DOI:** 10.1038/srep24272

**Published:** 2016-04-12

**Authors:** Xin Xu, Zhiyu Li, Luyao Cai, Sarah Calve, Corey P. Neu

**Affiliations:** 1 Department of Mechanical Engineering 1111 Engineering Drive, 427 UCB University of Colorado Boulder Boulder, CO 80309-0427, USA; 2 Purdue University Weldon School of Biomedical Engineering 206 South Martin Jischke Drive West Lafayette, IN 47907, USA.

## Abstract

The biomechanical properties of the extracellular matrix (ECM) play an important role in cell migration, gene expression, and differentiation. Biomechanics measurements of ECM are usually performed on cryotomed tissue sections. However, studies on cell/matrix interplay are impossible to perform due to disruptions in cell viability and tissue architecture from freeze-thaw cycling. We developed a technique to map the stiffness of living cells and surrounding matrix by atomic force microscopy and use fluorescence microscopy to relate those properties to changes in matrix and cell structure in embryonic and adult tissues *in situ*. Stiffness mapping revealed significant differences between vibratomed (living) and cryotomed tissues. Isolated cells are softer than those in native matrix, suggesting that cell mechanics are profoundly influenced by their three-dimensional environment and processing state. Viable tissues treated by hyaluronidase and cytochalasin D displayed targeted disruption of matrix and cytoskeletal networks, respectively. While matrix stiffness affected cellular stiffness, changes in cell mechanics did not reciprocally influence matrix stiffness.

Biological tissues are an ensemble of cells and extracellular matrix (ECM) that combine to carry out distinct functions within an organism. The reciprocal interactions between these two components are tightly regulated to facilitate proper tissue assembly during growth and to maintain homeostasis in the adult. The ECM is a biologically active scaffold that controls cell behavior by defining the stiffness of the environment and activating intracellular signaling via binding of specific cell-surface receptors^1^. Cells combine the signals from both ECM rigidity and composition and adjust their behavior and gene expression accordingly^2^,^3^. Conversely, cells are constantly remodeling the ECM, whether it is by changing the material properties during development through the addition and enzymatic removal of distinct components or by maintaining homeostasis through regulated turnover^4^. Perturbations in the local stiffness can alter gene expression, impacting differentiation, pathological behaviors and cancer progression^4^.

In order to resolve forces at the cellular level, researchers have utilized atomic force microscopy (AFM), a powerful tool for nanoscale imaging and force measurement. AFM has been widely applied in tissue biomechanics studies due to its ability to work in fluids and physiologically relevant environments^5^,^6^,^7^,^8^,^9^,^10^,^11^. However, current available methods require cells and ECM to be tested separately due to the size and complex architecture of most biological tissues. To circumvent the geometric constraints of intact tissues, cells are typically isolated from the native 3D tissue architecture and plated on artificial 2D substrates before analyzing via AFM^9^,^12^,^13^,^14^. While nano/micro-indentation by AFM has successfully revealed significant differences between cell types and states of differentiation^15^,^16^, complex interactions between the cell and ECM are neglected.

To measure ECM mechanics using AFM, frozen tissue sections are typically used^17^,^18^. Biological tissues are relatively soft, making them extremely difficult to cut into thin slices. The reduced temperature during cryosectioning significantly increases the tissue stiffness thus allowing for the preparation of semi-thin (5–50 μm thick) sections that can be easily deposited on a glass substrate for AFM scanning. However, cells cannot maintain viability at the low temperatures required for cryosectioning, making cell/matrix interaction studies impossible. Additionally, freeze-thaw cycles can greatly change the ECM architecture thus influencing tissue mechanical properties and response to treatment^19^,^20^. Therefore, maintaining tissue viability and architecture is an essential yet challenging need to properly study tissue and cell mechanics. While some studies have been able to capitalize on the small geometry and homogeneous properties of certain specimens to analyze fresh tissues with AFM^7^,^21^,^22^,^23^,^24^; none of these works directly measured the cell/ECM interactions.

Here, we demonstrate an AFM technique for sub-micron resolution mapping and comparison of biomechanical properties of cells and ECM within heterogeneous living tissues. Fluorescence microscopy was used to relate AFM measured compressive modulus with tissue structure. We found that the mechanical properties of both cells and ECM in living (vibratomed sections) versus dead (cryotomed sections) tissues were significantly different. Furthermore, we were able to show that perturbations in ECM composition can directly affect cell stiffness. Finally, we show that isolated cells were significantly less stiff than those embedded in native matrix, demonstrating the importance of maintaining the integrity of cell/matrix interactions during biomechanical studies.

## Results

### *In Situ* Biomechanical Properties of Living Cells in the Native Matrix of Embryonic and Adult Tissues

**Figure 1:**
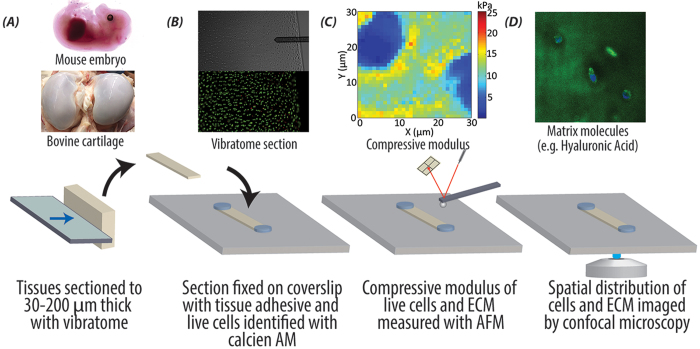
Combined AFM and fluorescence microscopy enables the study of biomechanical properties of individual cells and the surrounding ECM in live biological tissues. (**A**) Embryonic murine forelimbs or bovine cartilage plugs, obtained from medial anterior locations of bovine femoral condyles, were harvested and immediately sectioned with a vibratome. (**B**) Vibratomed sections were visualized with phase (top) and fluorescence microscopy (middle) and stained with a live (calcein-green)/dead (propidium iodide-red) assay, which indicated the majority of cells remained viable [70–90% viability]. Sections were fixed to coverslips using tissue adhesive applied away from tested regions. (**C**) The compressive modulus was mapped using force volume mode AFM at spatial resolutions (<1 μm/pixel) that enabled direct comparison of cell and matrix stiffness. (**D**) Confocal imaging was utilized to correlate compressive modulus with structural changes in cells and ECM.

By combining AFM and fluorescence microscopy, we were able to map stiffness of living cells and matrix, and relate those properties to the structure of native tissues *in situ*. We utilized fresh embryonic and adult tissues from common animal models (murine and bovine, respectively) and used a vibratome to generate thin sections (30–200 μm thick, Fig. 1A). Figure 1B shows a 30 μm thick adult bovine cartilage section that was stained with calcein AM to identify live cells, affixed at the ends with glue to a glass coverslip, then placed in a PBS-filled liquid cell for AFM. Approximately 70–90% cells remain viable after this procedure. The combination of the AFM with a fluorescence microscope allowed for the AFM probe to be carefully positioned at regions with live cells. Sub-micron resolution stiffness maps were acquired by force-volume mode AFM (Fig. 1C). After AFM testing, the tissues were fixed with 4% paraformaldehyde and fluorescently stained for different tissue components (Fig. 1D). AFM stiffness maps and fluorescent images of molecular components were then correlated to provide insight into cell/microenvironment interactions. Our results demonstrate that force volume mode AFM is a viable means for microscale mechanical property mapping of live biological tissues *in situ*.

### Vibratomed (Living) Cartilage is Significantly Stiffer than Cryotomed (Dead) Cartilage in the Murine Embryo

**Figure 2:**
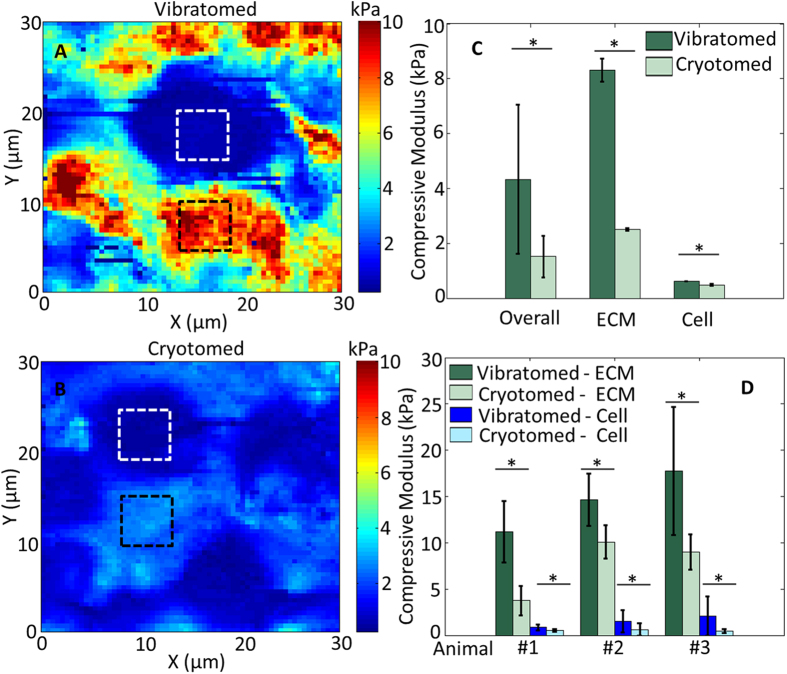
Freezing significantly alters the compressive modulus of embryonic tissues and prevents measurement of viable cells. Representative AFM modulus maps of the developing humerus in vibratomed (**A**) and cryotomed (**B**) sections of embryonic murine forelimbs (E18.5). (**C**) Comparison of overall (entire scan area), ECM (black squares) and cell (white squares) compressive moduli reveals freezing significantly reduces the stiffness of the matrix and cells in developing murine cartilage. (**D**) Statistical comparison of 3 different E18.5 embryos, 3 locations per animal. Two-way ANOVA reveals the vibratomed sections (ECM: 14.53 ± 5.42 kPa; cell: 1.51 ± 1.14 kPa) are significantly stiffer than the cryotomed sections (ECM: 7.61 ± 3.29 kPa; cell: 0.55 ± 0.41 kPa). *p < 0.0001.

Forelimb sections from the same embryo of embryonic day (E)18.5 were obtained using either a vibratome (200 μm thick) or a cryotome (5 μm thick). Due to the heterogeneity of tissue in the developing limb, it was not possible to match the thickness of vibratomed and cryotomed sections. Despite the difference in specimen thickness, we expect that there is negligible influence from the substrate since we used a very small indentation force of just 11.5 nN (corresponding to an average indentation depth of about 600 nm)^25^. Typical AFM compressive modulus maps of vibratomed and cryotomed sections reveal that the overall stiffness of the cryotomed embryonic tissue is significantly lower (Fig. 2). Cartilage is composed of chondrocytes surrounded by a pericellular matrix (PCM), containing perlecan and type VI collagen, which is distinct from the bulk ECM that is dominated by hyaluronic acid (HA) and type II collagen^26^. To quantitatively compare the cells and the ECM, we focused only on the stiffness of the bulk matrix, away from the PCM (as marked by the dashed squares in Fig. 2A,B). In mouse embryonic tissues, both the cellular regions and bulk ECM of the vibratomed section were significantly stiffer than the cryotomed section (p < 0.0001; Fig. 2D). Note that the cryotomed sections are only 5 μm thick, so the cells within the cryotomed sections are not intact.

### Vibratomed (Living) Cartilage is Significantly Softer than Cryotomed (Dead) Cartilage in Adult Bovine Cartilage

To test the capability of our technique to measure the mechanical properties of other tissues and to remove the variable of specimen thickness, the stiffness of vibratomed adult bovine cartilage was analyzed before and after freeze-thawing. For each animal, two vibratomed sections (30 μm thick) from the same cartilage plug were paired for comparison. First, both sections were scanned within 1 h after sectioning for control. Then one piece was incubated at 37 °C and 5% CO_2_ for about 12 h to maintain cell/tissue viability and scanned again. The other piece was frozen at −80 °C for about 12 h to promote the changes that may occur during cryosectioning, then thawed at 4 °C for at least 2 h and tested again. This procedure may not fully represent the cryosectioning case since embedding medium was not used here. Initial experiments performed on 30 μm thick cryosections resulted in measurements of 24.31 ± 4.75 kPa, suggesting that freezing increased sample stiffness. However, during subsequent scans, the modulus decreased to 4.92 ± 0.74 kPa (Supplementary Fig. S1). We attributed this inconsistency to the variable topography (e.g. tissue folds due to sample wrinkling) generated when obtaining cryosections >10 μm, which will spread out and relax with time. Therefore, to ensure any changes in mechanical properties are not due to sample variation, the comparison was made on the same tissue section.

**Figure 3:**
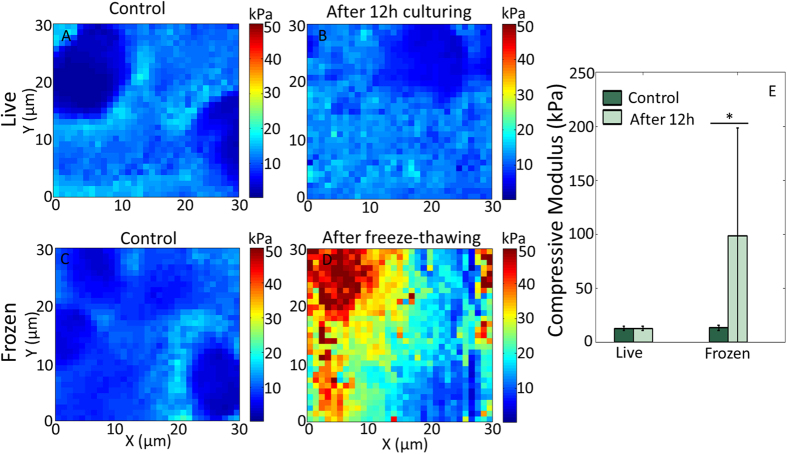
The compressive modulus of vibratome sections of adult bovine cartilage is maintained during live culturing but is significantly altered after freeze-thawing. AFM compressive modulus map of the same live bovine cartilage section scanned within 1 hour of sectioning (**A**) and after 12 hour incubation at 37 °C and 5% CO_2_ (**B**). Another viable sample sectioned at the same time as (**A**) was imaged before (**C**) and after (**D**) being frozen at −80 °C for 12 hours, thawed at 4 °C for 2 hours. (**E**) Statistical comparison of 3 different animals, 3 locations per animal. The compressive modulus of the vibratomed sections did not significantly change after 12 hours incubation (control: 12.63 ± 2.05 kPa; 12 h: 12.67 ± 1.97 kPa), while the frozen sections became significantly stiffer (control: 13.18  ± 2.25 kPa; 12 h: 98.64 ± 99.64 kPa. *p < 0.0001.

We observed the stiffness of live bovine cartilage sections did not change significantly after 12 h incubation (control: 12.63 ± 2.05 kPa; 12 h: 12.67 ± 1.97 kPa; p = 0.8552), whereas the sections after freeze-thawing were greatly stiffened (control: 13.18 ± 2.25 kPa; 12 h: 98.64 ± 99.64 kPa; p < 0.0001) (Fig. 3). Note the standard deviation of the cartilage stiffness after freeze-thawing (98.64 ± 99.64 kPa) is quite large, which is due to the extremely high stiffness (>600 kPa) at some regions.

Together, with the results from the embryonic tissues, our data demonstrate that the influence of freezing on tissue mechanics is variable, but significant.

### Hyaluronidase Treatment Increases the Stiffness of Both Bovine Cartilage ECM and Embedded Chondrocytes

**Figure 4:**
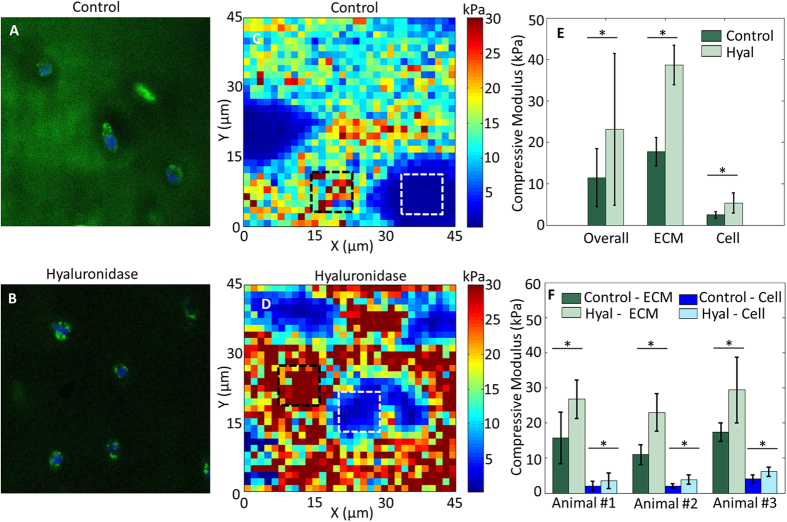
Targeted disruption of matrix composition by hyaluronidase alters both ECM and cell stiffness. Control (**A**) and hyal-treated (**B**) bovine cartilage stained with HABP shows an overall decrease in HA (green) throughout the tissue (blue = DAPI). Representative AFM compressive modulus maps of control (**C**) and hyal-treated (**D**) bovine cartilage sections. (**E**) Comparison of overall (entire scan area), ECM (black squares) and cell (white squares) compressive moduli reveals removal of HA increases both ECM and cell stiffness. (**F**) Statistical comparison of 3 different animals, 3 locations per animal. Hyal treatment significantly stiffened both ECM (14.71 ± 5.49 kPa before and 26.40 ± 7.45 kPa after) and cells (2.69 ± 1.44 kPa before and 4.51 ± 2.02 kPa after). *p < 0.0001.

To assess whether our technique is sensitive enough to measure perturbations in ECM composition, we treated vibratomed cartilage with hyaluronidase (Hyal) to disrupt the HA network. Fresh bovine cartilage sections were tested before and after 30 min digestion with 7,500 U/mL Hyal in PBS. Before Hyal digestion, HA (green) was present ubiquitously throughout the ECM especially in the PCM (Fig. 4A; DAPI = blue), consistent with previous reports^27^. After Hyal digestion, HA staining was substantially reduced in the PCM and was difficult to observe in the bulk ECM area as well (Fig. 4B). Even though the HA component was significantly reduced, most chondrocytes maintained viability with no obvious difference observed via calcein AM staining before and after Hyal treatment. Hyal-mediated removal of HA resulted in a significant increase in bulk ECM modulus (*n* = 3 animals; p < 0.0001; Fig. 4C–F). Interestingly, the cell modulus also increased after Hyal treatment (p < 0.0001; Fig. 4E,F).

### Cytochalasin D Decreases the Stiffness of Matrix-Embedded Bovine Chondrocytes, but not Surrounding Cartilage ECM

**Figure 5:**
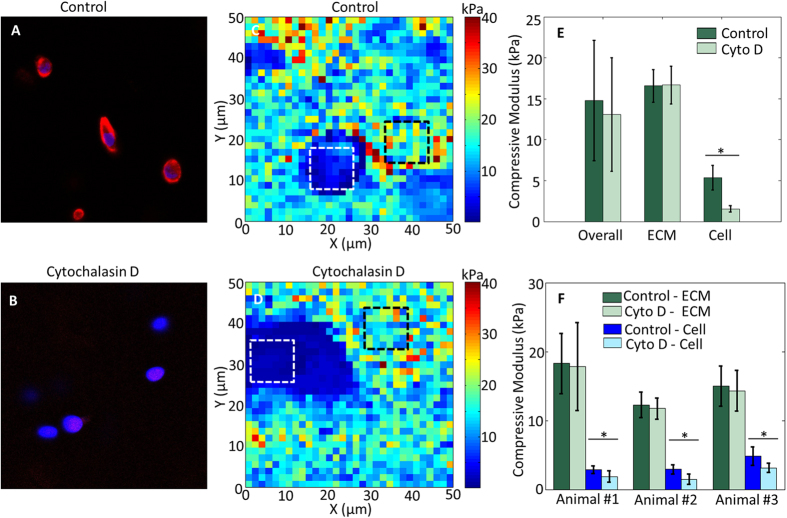
Disruption of the actin cytoskeleton significantly alters cell but not ECM compressive modulus. Control (**A**) and CytoD-treated (**B**) bovine cartilage stained with phalloidin shows an overall decrease in actin (red) throughout the embedded chondrocytes (blue = DAPI). Representative AFM compressive modulus maps of control (**C**) and CytoD-treated (**D**) bovine cartilage sections. (**E**) Comparison of overall (entire scan area), ECM (black squares) and cell (white squares) compressive moduli in panels (**C**,**D**) reveals actin network disruption only affects cell stiffness. (**F**) Statistical results of 3 different animals, 3 locations per animal. Cyto D treatment significantly softened the cells (3.54 ± 1.29 kPa before and 2.16 ± 1.04 kPa after) but not the ECM (15.17 ± 4.03 kPa before and 14.64 ± 4.85 kPa after). *p < 0.0001.

To determine if matrix composition predominates over cytoskeletal architecture as a determinant of cell stiffness, the actin network was disrupted with cytochalasin D (CytoD), which was expected to leave the ECM intact. Before CytoD treatment, the filamentous actin (red) within the chondrocytes was intact (Fig. 5A; DAPI = blue). After being treated with 0.5 mg/mL CytoD for 30 min, the actin cytoskeleton was largely disrupted (Fig. 5B). CytoD treatment significantly decreased the compressive modulus of the chondrocytes (p < 0.0001); however, there was no change in the bulk ECM (p = 0.339, *n* = 3 animals, Fig. 5C–F).

### Isolated Chondrocytes are Softer than Those in Native Tissue Matrix

**Figure 6:**
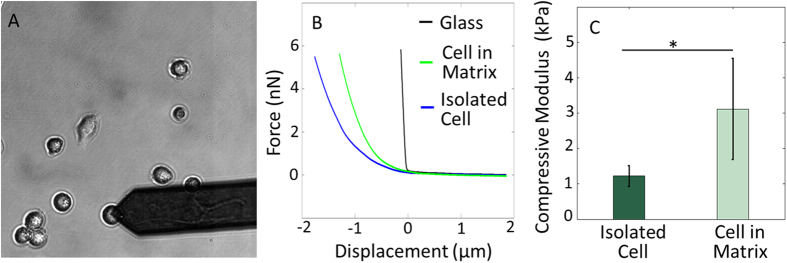
The *in situ* compressive modulus of cells is not maintained after isolation. (**A**) Primary chondrocytes from bovine cartilage were tested 2 hours after isolation. (**B**) Typical AFM force-distance curves (loading) obtained on glass, chondrocyte in native matrix and isolated chondrocyte. (**C**) Isolated chondrocytes (1.22 ± 0.29 kPa; n = 15) are significantly softer than those in their native matrix (3.11 ± 1.42 kPa; n = 6). *p < 0.0001.

The current paradigm for measuring individual chondrocyte stiffness is to isolate cells enzymatically from the native tissue and plate them on a culture dish or a glass slide prior to measurement by AFM^15^,^28^. To assess our technique with the standard means of measuring cell stiffness, and to quantify how interactions with the native matrix directly influences chondrocyte mechanics, we compared the stiffness of chondrocytes within fresh vibratomed sections with those isolated by collagenase P digestion (Fig. 6A). Typical AFM indentation curves (force-distance curves) on chondrocytes within or isolated from the native matrix are shown in Fig. 6B. The compressive modulus of chondrocytes was 1.22 ± 0.29 kPa (n = 15) 2 hours after isolation, which is significantly softer than cells measured within vibratomed sections (in Figs 4 and 5) 3.11 ± 1.42 kPa (p < 0.0001; Fig. 6C).

## Discussion

We demonstrated a novel technique for sub-micron resolution mapping of the biomechanical properties of cells and ECM within living tissues. An important finding of our study was that maintaining tissue viability makes the study of microenvironment/cell interactions possible. The careful sample preparation for thin (vibratomed) sections, with embedded viable cells, will enable the unique combination of atomic force microscopy with optical microscopy. Although our current studies were limited to widefield microscopy for cell viability assessments, future studies can be readily extended to map the distribution of a myriad of specific intra- and extracellular markers using immunofluorescence labeling. In addition, the study of sample sections within an aqueous environment (with chemically-defined medium) permitted the application of reagents to selectively target components of the matrix (e.g. disruption of HA) or cytoskeleton (e.g. disruption of actin polymerization), opening up numerous possible small-scale, controlled studies of cartilage degeneration through the pathogenesis of osteoarthritis.

The power of our technique comes from the ability to perturb cell or ECM biology and directly measure the effects of tissue micromechanics. By disrupting and removing HA by Hyal, we were able to study the influence of altered matrix stiffness on embedded cells. The increase in the bulk ECM modulus of fresh cartilage after Hyal digestion is possibly due to the predominance of the stiffer type II collagen fibrils after the removal of HA^29^. Our result is contrary to a previous study wherein the stiffness of 5 μm thick cryotomed cartilage sections treated by 60 U/mL Hyal solution was measured by AFM^18^. The possible discrepancy in the results could be that we used a 125-fold higher concentration of Hyal and/or that freeze-thaw cycling changed the ECM architecture of the cryosections thus changing the ability of the ECM to respond to both swelling upon rehydration in PBS and the biochemical treatments. Indeed, macroscale testing of fresh cartilage explants showed that the storage modulus increased after 1 hour of Hyal incubation^30^, a similar trend to what we found in our study.

Utilizing the same incubation parameters, we disrupted actin polymerization and demonstrated that our technique has the sensitivity to measure how treatments can differentially affect the cell and surrounding ECM. We observed low levels of phalloidin staining after the CytoD treatment; however, most of the chondrocytes were still viable (via calcein stain). The AFM measurement shows that the chondrocytes were softer after the CytoD digestion, while we did not observe significant change in the ECM stiffness, which is expected as CytoD is known to alter the organization of actin cytoskeleton and thus reduces cell stiffness without affecting the matrix. The absence of any change in matrix stiffness due to CytoD strengthens our results obtained after Hyal digestion since the diluent and incubation parameters were the same for the two treatments. This same approach could be applied to many other cell types (e.g. myocytes, hepatocytes, neurons) and treatments (e.g. enzymatic digestions, chemotherapy exposure) to quantify the interplay between cells and the microenvironment.

Our results further indicate that freeze-thawing of samples profoundly altered tissue stiffness with disparate trends for thin (decreasing stiffness) and thick tissue sections (increasing stiffness). The results are significant because for tissue storage or easy manipulation, many tissue samples prepared for mechanics testing have undergone at least one freeze-thaw cycle. But the freeze-thaw process likely alters the ECM architecture, thus changing tissue structure and material properties. Our results emphasize that in order to accurately measure the micromechanical properties of biological tissues, it is necessary and feasible to study viable samples.

Our results for the stiffness of cryosectioned bovine articular cartilage and isolated chondrocytes are in line with previously published reports^31^,^32^,^33^. The modulus of bovine articular cartilage after freeze-thawing was reported to be about 0.7 MPa using a 50 μm radius indenter^31^. The modulus of isolated adult bovine chondrocytes was found to be about 1 kPa using micropipette aspiration method^32^, or about 3 kPa when relaxed in 2% alginate^33^. Furthermore, the difference in fresh vs. frozen murine embryo samples is consistent with published results on the macroscale testing on bovine and porcine articular cartilage^34^,^35^. The reduction in compressive modulus of the embryonic specimens is possibly due to the increase in matrix pore size as a result of ice crystal formation in cryopreservation, or disruption of ECM networks during freezing^36^.

Most cell types are capable of sensing and responding to small changes in the mechanics of their microenvironments^37^. Therefore, bioengineers have been characterizing the mechanical properties of biological tissues with the aim of using this information as design parameters for physiologically-relevant *in vitro* environments and scaffolds for tissue repair. However, as demonstrated by work from our laboratories and others, the measured stiffness of biological tissues is significantly affected by specimen handling such as freezing. By combining AFM stiffness maps of cells and matrix with fluorescent microscopy-based measures of structure, we developed a feasible technique to study the mechanical properties of viable embryonic and adult tissues. Careful preparation of tissue samples, including maintenance of the temperature above 0 °C, is needed for the accurate study of cell-matrix interactions at the nanoscale. We found that while tissue matrix stiffness affects cellular stiffness, changes in cell mechanics do not reciprocally influence matrix stiffness. Even though our results were obtained using cartilage sources from murine embryos and bovine stifles, we expect that this technique will be applicable to a wide range of biological tissues, including brain and skin, in addition to active (heart, skeletal muscle) and passive (ligament, hydrogel) biomaterials.

## Methods

### Tissue Acquisition

Embryonic day (E)18.5 embryos were harvested from time-mated wild type C57Bl6/J mice obtained from the Jackson Laboratory (Bar Harbor, ME, USA). All murine experiments were approved by the Purdue Animal Care and Use Committee (PACUC protocol 1209000723). PACUC ensures that all animal programs, procedures, and facilities at Purdue University adhere to the policies, recommendations, guidelines, and regulations of the USDA and the United States Public Health Service (USPHS) in accordance with the Animal Welfare Act and Purdue’s Animal Welfare Assurance. Dams were euthanized via CO_2_ inhalation, which was confirmed by cervical dislocation, and the embryos were removed from the uterine horns in sterile phosphate-buffered saline (PBS; Life Technologies, Grand Island, NY, USA).

To compare mechanical properties of viable, fresh (vibratomed) tissue and frozen (cryotomed) tissue, forelimbs from the same embryo were paired for each set of data. For vibratome sectioning, the forelimbs were carefully removed and coated with tissue adhesive (Electron Microscopy Sciences, Hatfield, PA, USA), then embedded in in 4% low melt agarose (BIO-RAD, Hercules, CA, USA) (Fig. 1A). The agarose-embedded forelimbs were sliced into 200 μm sections (Fig. 1B) by a Leica VT-1000S vibratome (Leica Microsystems Inc., Germany). To maintain agarose rigidity and hydration while vibratoming, the embedded specimens were placed in a PBS-filled water bath surrounded with ice and the cut sections were kept on ice before testing to preserve cellular and matrix integrity. For AFM testing, the slices were washed thoroughly with PBS and incubated in Dulbecco’s modified Eagle’s medium (DMEM; Life Technologies, Grand Island, NY, USA) with calcein for 20 min to identify live cells. Calcein AM (Life Technologies, Grand Island, NY, USA) was dissolved in dimethyl sulfoxide (DMSO; Sigma-Aldrich, St. Louis, MO, USA) at a concentration of 1 μg/μL, and then the solution was added to DMEM at a ratio of 1:1000. The calcein-stained section was then placed on a coverslip coated with a thin layer of tissue adhesive. The coverslip was placed in a liquid cell filled with PBS and ready for AFM test. Care was taken to keep specimens hydrated at all time points.

For cryotome sectioning, the embryonic forelimbs were embedded in clear frozen section compound (VWR International, Radnor, PA, USA), frozen in dry ice-cooled isopentane and stored at −80 °C until sectioning. A Shandon Cryotome™ FE Cryostat (Thermo Scientific Inc., Waltham, MA, USA) was used to transversely slice 5 μm thick sections of the embryonic forelimbs. The sections were then collected on a glass coverslip and washed thoroughly with PBS to remove the clear frozen section compound prior to AFM testing. Importantly, even though the cryotomed sections are much thinner than the vibratomed sections, they are thick enough to overcome any influence from the stiffness of the substrate^25^ since we used a very small indentation force of just 11.5 nN (corresponding to an average indentation depth of about 600 nm, or only ~12% of the sample thickness).

Articular cartilage was harvested from juvenile bovine knee (stifle) joints obtained from a local United States Department of Agriculture (USDA) regulated abattoir (Dutch Valley Foods, Holland, IL, USA) within 36 hours of slaughter. As these joints were classified as non-edible food items, no additional regulatory compliance was necessary. Joints were opened under aseptic conditions to expose the tibial and femoral condyles and the trochlear groove. Osteochondral (cartilage-bone) plugs were removed from the load-bearing region of the distal femoral condyles with an 8mm diameter coring reamer. The plugs were then sliced perpendicular to the articular surface in 30 μm thick sections by a Leica VT-1000S vibratome within 2 hours of harvest, as described above. Cartilage sections were washed thoroughly with PBS and incubated in culture medium consisting of DMEM with 10% fetal bovine serum (Life Technologies, Grand Island, NY, USA), 1% bovine serum albumin (Sigma-Aldrich, St. Louis, MO, USA) and 1% penicillin and streptomycin (Life Technologies, Grand Island, NY, USA) at 37 °C and 5% CO_2_ before AFM testing. Cartilage sections were then pre-stained with calcein to identify live cells, and affixed to a coverslip by putting a tiny drop of cyanoacrylate (Loctite, Westlake, OH, USA) to the ends of the sections but not the testing regions (Fig. 1B). Again care was taken to keep specimens hydrated at all time points.

### Bovine Chondrocytes

Osteochondral plugs were obtained as described above. The superficial zone (~200 μm) and deep zone (near the subchondral bone) were carefully removed from plugs with a custom cutting jig. Then the remaining middle zone cartilage (~300 μm thick) was washed twice in sterilized PBS and chopped into fine fragments. These fragments were collected and immersed in 0.1% (wt/vol) collagenase P (Roche Diagnostics GmbH, Mannheim, Germany) in DMEM at 37 °C with agitation. After 2–3 hour digestion, the cell solutions were filtered by 70 μm Nylon cell strainer (Falcon, Waltham, MA, USA), and then centrifuged and resuspended twice in PBS. The final cell populations were seeded onto petri dish coated with poly-L-lysine (PLL) (Sigma-Aldrich, St. Louis, MO, USA). After 2 hour incubation at 37 °C, chondrocytes formed a strong attachment to the petri dish while retaining a rounded morphology and were ready for AFM testing. Note this test was intentionally done on cells before they have a chance to fully spread out^15^,^28^ and take on a more physiologically irrelevant fibroblastic phenotype^38^, to mimic the rounded morphology of chondrocytes *in situ*.

### Mechanical Characterization via AFM Stiffness Mapping

A Keysight 5500 AFM system (Keysight Technologies Inc., Santa Rosa, CA, USA) was combined with a Nikon Eclipse Ti wide-field inverted microscope (Nikon Instruments Inc., Melville, NY, USA), allowing for simultaneous AFM scanning and fluorescence microscopy. For tissue sections, the AFM was operated in force volume mode, wherein an array (32 × 32 or 64 × 64 points) of force-distance (F–Z) curves was collected over the entire scan area. For isolated chondrocytes, the AFM probe was carefully placed on top of each individual cell for testing, and three sets of F–Z curves were collected for each cell. The force trigger was set to about 11.5 nN indicating the point at which the cantilever approach was stopped and then retracted. By fitting the F–Z curves to a contact mechanics model (i.e. Hertzian^39^), the compressive modulus was extracted. Thus the force volume mode imaging provides a map of the sample material properties simultaneously with the topography map (Fig. 1C). To ensure accurate model fitting, a known AFM tip geometry is essential. A cantilever with a 5 μm borosilicate glass sphere attached to the free end (NovaScan, Ames, IA, USA) was used. The cantilever stiffness was pre-calibrated to be 0.07 N/m by the thermal fluctuation method^40^.

### Hyaluronidase and Cytochalasin D Treatment of Bovine Cartilage

Hyaluronidase (Hyal; Worthington Biochemical Corp., Lakewood, NJ, USA) was diluted to 7,500 U/mL in sterile PBS. Bovine cartilage sections were incubated in 50 μL of 7,500 U/mL Hyal solution for 30 min. Hyaluronic acid (HA) digestion was confirmed by fluorescent localization of HA using a biotinylated Hyaluronic Acid Binding Protein (HABP; Calbiochem, Billerica, MA, USA). Control and Hyal-incubated sections were labeled with HABP (1:300) and streptavidin-488 (1:500; Life Technologies, Grand Island, NY, USA) to visualize HA content and DAPI (1:500; Roche, Mannheim, Germany) to identify nuclei.

Cytochalasin D (Cyto D; 5 mg/mL in dimethyl Sulfoxide, Sigma-Aldrich, St. Louis, MO, USA) was diluted to 0.5 mg/mL in PBS. 50 μL of the diluted CytoD was used to treat the bovine cartilage for 30 min. Filamentous actin disruption was confirmed by fluorescence microscopy for phalloidin-555 (1:100; Life Technologies, Grand Island, NY, USA) on undigested control and digested sections, in addition to DAPI (1:500) to identify cell locations.

Control and treated sections were imaged with a Zeiss LSM 710 microscope at Purdue University’s Life Science Fluorescence Imaging Facility using a 63× oil objective. All the imaging parameters were exactly the same for the control and treated sections.

### Statistical Analysis

For statistical analyses, each treatment was performed on three animals, and each animal was evaluated at 3 different locations. For each location, a 5 × 5 pixel^2^ matrix was taken from the AFM stiffness map at the middle of a cell and matrix region, respectively, to calculate the average cell and ECM stiffness. The p-value was calculated using IBM SPSS Statistics (IBM Corp., Armonk, New York, USA).

## Additional Information

**How to cite this article**: Xu, X. *et al.* Mapping the Nonreciprocal Micromechanics of Individual Cells and the Surrounding Matrix Within Living Tissues. *Sci. Rep.* **6**, 24272; doi: 10.1038/srep24272 (2016).

## Supplementary Material

Supplementary Information
