# Supplementary material for: Mapping the Nonreciprocal Micromechanics of Individual Cells and the Surrounding Matrix Within Living Tissues
Source: Sci Rep. 2016 Apr 12;6:24272. doi: 10.1038/srep24272 (PMC4828668; doi:10.1038/srep24272)
Supplement: Supplementary Information [file srep24272-s1.doc]

***Mapping the Nonreciprocal Micromechanics of Individual Cells and the Surrounding Matrix Within Living Tissues***

Xin Xu

Zhiyu Li

Luyao Cai

Sarah Calve

Corey P. Neu

**SUPPLEMENTARY INFORMATION**


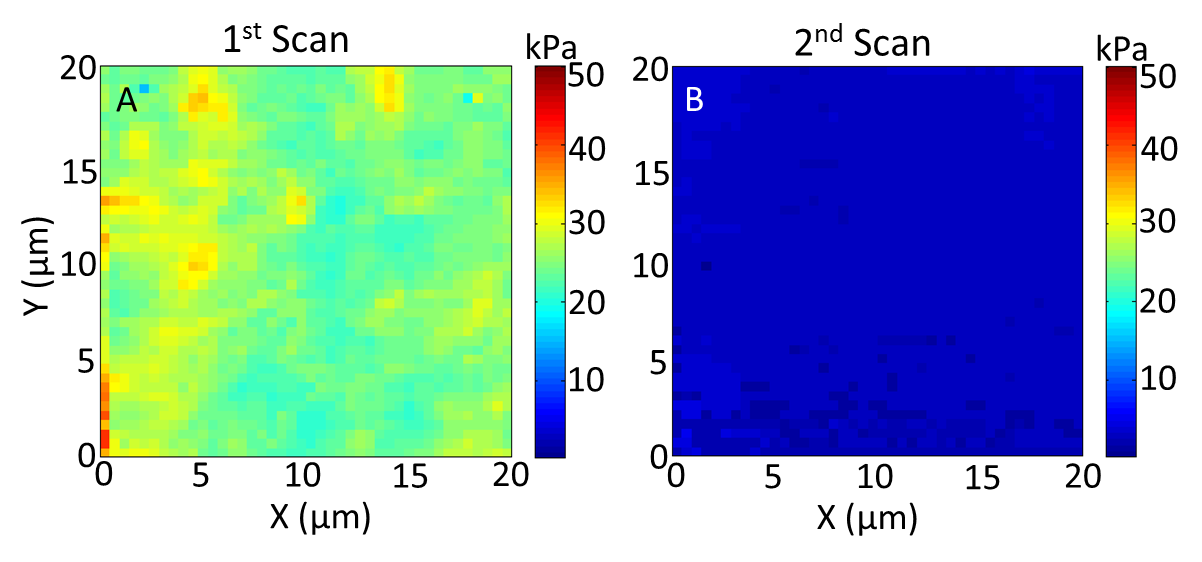


**Supplementary Figure S1.** AFM compressive modulus maps of a 30 μm thick cryosection revealed (A) initial measurements of 24.31±4.75 kPa, suggesting that freezing increased sample stiffness. However, (B) during subsequent scans within 20 mins, the modulus decreased to 4.92±0.74 kPa.
